# Risk factors of a severe course of pediatric multi-system inflammatory syndrome temporally associated with COVID-19

**DOI:** 10.1007/s00431-022-04584-8

**Published:** 2022-08-10

**Authors:** Aleksandra Stasiak, Ewelina Perdas, Elżbieta Smolewska

**Affiliations:** 1 Department of Pediatric Cardiology and Rheumatology, Medical University of Lodz, Sporna 36/50 street, 91-738 Lodz, Poland; 2 Department of Biostatistics and Translational Medicine, Medical University of Lodz, Lodz, Poland

**Keywords:** SARS-CoV-2, PIMS, Child, Risk factors

## Abstract

Pediatric multi-system inflammatory syndrome temporally associated with COVID-19 (PIMS-TS) is a serious complication of a previous SARS-CoV-2 infection. The disease causes multiple organ failure, but in some patients, a more severe course of the disease is observed. The treatment is multidirectional and depends on the severity and course of the disease, as some patients do not respond to the recommended treatment. The aim of this study was to identify laboratory risk factors affecting the more severe course of the disease and resistance to standard therapy. It is a single-center retrospective study considering 51 patients with PIMS-TS. Clinical features, laboratory results, and additional imaging tests data were taken into account. Fifty-one patients with PIMS-TS were hospitalized within a 16-month observation period. In the studied group, 26/51 children (51%) were girls. The mean age of patients was 7 years. Sex of the patient was not a risk factor for changes in cardiovascular system or severe course of the disease. Sixteen patients (31.3%) required transfer to the intensive care unit. Children with initially higher concentrations of NT-proBNP, troponin, creatinine, triglycerides, C-reactive protein, procalcitonin, ferritin, D-dimers and lower hematocrit, platelet count, lymphocytes, and ejection fraction should be strictly observed as they have a higher risk of severe course of the disease.

*Conclusions*: Laboratory parameters especially markers of myocardial damage, markers of inflammation, blood count, as wells as biochemical parameters are significant risk indicators of severe course of PIMS -TS and their concentration can be defined as predictor of disease severity.

**Table:** 

**What is Known:** *• Pediatric multisystem inflammatory syndrome temporally associated with COVID-19 (PIMS-TS) is a serious complication of a previous SARS-CoV-2 infection in the group of pediatric patients* *• Course of the disease may be severe, which may cause long-term complications and the need for longitudinal patient care.*	
**What is New:** *• Children with higher concentrations of NT-proBNP, troponin, creatinine, TG, CRP, PCT, ferritin, D-dimers and lower hematocrit, PLT, lymphocytes, and EF have a higher risk of a severe course of the disease.* *• Patients with high concentration of NT-proBNP, troponin, CRP, lactates, ferritin, D-dimers, creatinine and a lower concentration of PLT, albumin, leukocytes; lymphopenia, hyponatremia are at risk for intravenous immunoglobulin resistance.*

**What is Known:**

*• Pediatric multisystem inflammatory syndrome temporally associated with COVID-19 (PIMS-TS) is a serious complication of a previous SARS-CoV-2 infection in the group of pediatric patients*

*• Course of the disease may be severe, which may cause long-term complications and the need for longitudinal patient care.*

**What is New:**

*• Children with higher concentrations of NT-proBNP, troponin, creatinine, TG, CRP, PCT, ferritin, D-dimers and lower hematocrit, PLT, lymphocytes, and EF have a higher risk of a severe course of the disease.*

*• Patients with high concentration of NT-proBNP, troponin, CRP, lactates, ferritin, D-dimers, creatinine and a lower concentration of PLT, albumin, leukocytes; lymphopenia, hyponatremia are at risk for intravenous immunoglobulin resistance.*

## Introduction

Pediatric multi-system inflammatory syndrome temporally associated with COVID-19 (PIMS-TS), which is also referred to as multisystem inflammatory syndrome is a hyperinflammatory condition affecting multiple organs and leading to symptoms of shock in children and adolescents who previously contracted SARS-CoV-2 infection [1, 2]. According to the World Health Organization PIMS-TS syndrome can be diagnosed in children and adolescents between 0 and 19 years presenting with fever, clinical symptoms from ≥ 2 organ systems, elevated markers of inflammation, with no other obvious cause of inflammation, and evidence of SARS-CoV-2 infection in the last 4–8 weeks. Symptoms of the disease may be similar to the course of Kawasaki disease or toxic shock. Treatment includes intravenous immunoglobulins (IVIG), systemic corticosteroids, and in some cases biological therapy [2–4]. Some of the patients develop a more severe course of the disease with hypotension and cardiovascular failure leading to the necessity of treatment in the pediatric intensive care unit.

## Material and methods

The objective of this study was to identify laboratory risk factors affecting the more severe course of the disease and resistance to standard therapy. Demographic data (sex, age), clinical features, laboratory tests results, and additional imaging tests data were taken into account. Information was acquired from database of patients hospitalized in the single Polish center from June 2020 to October 2021. The study was performed according to the standard of the Helsinki Declaration. Legal guardians of patients consented to review of the medical records and to the use of the data. Diagnosis was based on clinical and laboratory criteria according to the World Health Organization/American Academy of Pediatrics statements. Clinical features, laboratory results, and treatment were evaluated. All the laboratory tests were performed by the standard methods.

For statistical analysis, the Shapiro–Wilk test was performed to test for normal distribution. Continuous variables are presented as median with the values of the lower and upper quartiles (25–75 percentile). The nonparametric Mann–Whitney *U* test was used for comparison between two groups. Categorical​ variables are presented as numbers with an appropriate percentage. The chi-square test with appropriate corrections applied depending on the size of the subgroups was used for their analysis. Correlations were assessed using the Spearman’s rank correlation coefficient. All *p*-values ≤ 0.05 were considered statistically significant. Statistically significant differences between the groups are presented graphically in the charts. Statistical analysis was performed using the Dell Statistica 13 data analysis software (StatSoft Polska, Krakow, Poland).

## Results

From June 2020 to October 2021, 51 patients with PIMS-TS (26 boys and 25 girls) were hospitalized. The median age of the patients was 7 years, (min. 3 months; max. 17 years). Forty patients were diagnosed as a regular PIMS-TS, 9 as Kawasaki-like PIMS-TS, and 2 as macrophage activation syndrome in the course of PIMS-TS. In our study, we took into account age, sex, number of days of fever, and certain laboratory parameters (i.e., peripheral blood count, markers of inflammation, heart failure enzyme levels), as well as echo- and electrocardiographic parameters.

Within 51 patients, 86% had positive antibodies for SARS-CoV-2 infection, whilst 14% had a positive result of RT-PCR test for SARS-Cov-2 infection in addition to other criteria of PIMS-TS. The most frequently presented clinical symptoms of PIMS-TS were fever (51/51), cardiovascular abnormalities (51/51), respiratory symptoms (38/51), gastrointestinal symptoms (27/51), rash (25/51), arthritis (7/51), and neurological symptoms (6/51) patients.

**Fig. 1:**
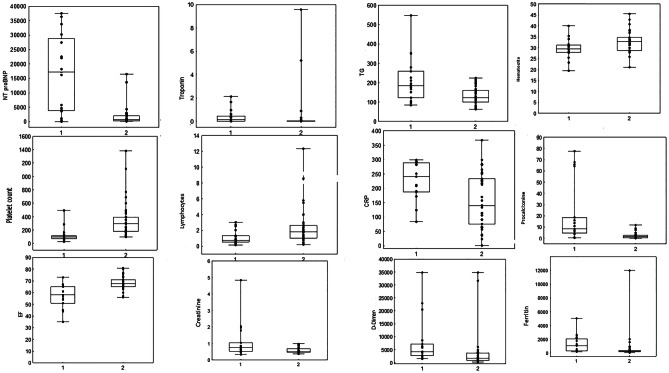
Parameters correlating with the more severe course of the disease (transfer of the child to the ICU). 1—patients who required ICU care; 2—patients who did not require ICU care*.* CRP, C-reactive protein; EF, ejection fraction; TG, triglycerides

Sixteen out of fifty-one children required a transfer to the intensive care unit (ICU). In the course of our study, we discovered that the parameters, which seem to be risk indicators of a more severe course of PIMS-TS associated with hospitalization in the ICU and thus prolonged hospitalization, were N-terminal pro-brain natriuretic peptide (NT-proBNP) (*p* = 0.0001), troponin (*p* = 0.0012), creatinine (*p* = 0.0128), triglycerides (TG) (*p* = 0.0115), hematocrit (*p* = 0.0272), platelet count (PLT) (*p* = 0.0000), lymphocytes (*p* = 0.0240), C-reactive protein (CRP) (*p* = 0.0132), procalcitonin (PCT) (*p* = 0.0001), ferritin (*p* = 0.0006), D-dimers (*p* = 0.0022), and ejection fraction (EF) (*p* = 0.0001) (Fig. 1).

Parameters that influenced the risk of respiratory failure, and therefore the need for mechanical ventilation (13/51), were NT-proBNP (*p* = 0.007), troponin (*p* = 0.0016), high-density lipoprotein (HDL) (*p* = 0.0405), lymphocytes (*p* = 0.0246), hemoglobin (*p* = 0.0095), hematocrit (*p* = 0.0032), PLT (*p* = 0.0004), PCT (*p* = 0.013), ferritin (*p* = 0.0020), D-dimers (*p* = 0.0019), and EF (*p* = 0.0014).

Parameters that influenced the risk of circulatory failure, hypotension (17/51) and therefore the need for administration of catecholamines (17/51), were hematocrit (*p* = 0.0272), lymphocytes (*p* = 0.0240), PLT (*p* = 0.0000), CRP (*p* = 0.0132), PCT (*p* = 0.0001), ferritin (*p* = 0.0006), D-dimers (*p* = 0.0022), TG (*p* = 0.0115), creatinine (*p* = 0.0128), NT-proBNP (*p* = 0.0001), troponin I (*p* = 0.0012), and EF (*p* = 0.0001).

Forty-five out of fifty-one patients received IVIG. Twenty-three of them required additional administration of systemic corticosteroids. Due to the severe course of the disease, two patients required administration of cyclosporine, and three required biological therapy with tocilizumab (a humanized anti-interleukin-6 receptor antibody, and one patient received anakinra (anti-interleukin-1 receptor antagonist). The risk factors for resistance to the administration of IVIG were analyzed. Statistical significance was obtained for parameters such as high concentration of NT-proBNP (*p* = 0.0110), troponin (*p* = 0.0023), CRP (*p* = 0.0377), lactates (*p* = 0.0206), ferritin (*p* = 0.0014), D-dimers (*p* = 0.0008), and creatinine (*p* = 0.0026) and a lower concentration of PLT (*p* = 0.0000), albumin (*p* = 0.0037), leukocytes (*p* = 0.0348), lymphopenia (*p* = 0.0160), and hyponatremia (*p* = 0.0186) (Table 1). Parameters that influenced the need to add systemic steroids to the treatment regimen were NT-proBNP (*p* = 0.0086), troponin (*p* = 0.0283), creatinine (*p* = 0.0200), TG (*p* = 0.0180), lactate (*p* = 0.00173), sodium (*p* = 0.00215), albumin (*p* = 0.0023), lymphocytes (*p* = 0.0087), PLT (*p* = 0.0000), CRP (*p* = 0.0056), PCT (0 = 0.0000), ferritin (*p* = 0.0003), D-dimers (*p* = 0.0001), and EF (*p* = 0.0001). In the performed statistical analysis, it was not found that any of the parameters significantly influenced the necessity to administer biological or immunosuppressive treatment, such as cyclosporine.

**Table 1:** Statistically significant indicators for immunoglobulin resistance in children with PIMS-TS

**Variable**	**Valid** ***N*** **Group 1**	**Median**	**25% quartile**	**75% quartile**	**Valid** ***N*** **Group 2**	**Median**	**25% quartile**	**75% quartile**	***p*-value**
NT-proBNP [pg/ml]	**21**	985	249	2544	**20**	5201	685	24,981	**0.0110**
Troponin I [ng/ml]	**22**	0.006	0.001	0.014	**20**	0.051	0.014	0.259	**0.0023**
Platelet count [thou/ul]	**24**	309	226	484	**22**	108	86	160	**0.0000**
Leukocytes [thou/ul]	**24**	11.44	10.20	18.23	**22**	8.41	5.42	13.50	**0.0348**
Lymphocytes [thou/ul]	**24**	1.99	0.99	2.98	**22**	0.82	0.54	1.45	**0.0160**
CRP [mg/l]	**24**	140	91	253	**22**	219	172	289	**0.0377**
Procalcitonin [ng/ml]	**24**	1.41	0.42	1.92	**22**	6.63	3.70	16.05	**0.0000**
Lactates [mmol/l]	**19**	1.68	1.00	2.19	**21**	2.49	1.76	3.01	**0.0206**
Sodium [mmol/l]	**24**	138	136	140	**22**	136	132	137	**0.0186**
Ferritin [ug/l]	**24**	226	130	400	**22**	578	296	1729	**0.0014**
D-Dimers [ng/ml]	**24**	1758	856	3579	**21**	4240	2638	6301	**0.0008**
Creatinine [mg/dl]	**23**	0.48	0.45	0.56	**22**	0.67	0.53	0.85	**0.0026**
Albumin[g/dl]	**23**	3.50	3.00	3.90	**22**	2.90	2.70	3.00	**0.0037**
EF [%]	**17**	58	51	65	**34**	67.5	65	71	**0.0001**

Group 1, patients who only required administration of IVIG; group 2, patients who required additional treatment (steroids, biological agents)

*CRP* C-reactive protein, *EF* ejection fraction

Apart from ionic disturbances, most of the patients had very low serum concentration of vitamin D3-OH-25. The concentration of vitamin D3 in children with PIMS negatively correlated with the concentration of NT-proBNP, troponin, D-dimers, and the age of the patients.

## Discussion

PIMS-TS is a relatively new and not yet fully understood condition. From the clinical experience and literature, it is known that PIMS-TS is a group of clinical and laboratory symptoms that occur in children after usually mild or asymptomatic SARS-CoV-2 infection. PIMS-TS most often causes multi-organ damage and even leads to shock [5]. In the literature, the average age of children suffering from PIMS-TS is 8 years and male sex seems to be slightly more predisposing [1]. In our study group, the mean age of the patients was 7 years, and the gender distribution was equal. The etiology of this syndrome is not yet fully understood, but it seems that genetically susceptible patients develop PIMS-TS as a result of a cytokine storm after the infection [5, 6].

In our study group, we did not obtain a statistically significant influence of the gender of children on the course of the disease; however, older children (> 7 years of age) had a greater risk of changes in the cardiovascular system (electrocardiographic abnormalities).

The treatment of the disease is multidirectional and depends on the severity and course of the disease. In accordance with current guidelines, first-line anti-inflammatory therapy for all children with PIMS-TS is IVIG. Second-line therapy is intravenous methylprednisolone, and it is considered for children who remain unwell 24 h after infusion of IVIG, particularly if they have ongoing fever. Biological therapy (IL-1R antagonists, IL-6 receptor blockers, or anti-TNF agents) should be considered a third-line option [7]. In our analysis, we investigated whether there are risk factors for resistance to treatment with IVIG and steroids in children with PIMS–TS. The analysis showed that a higher concentration of cardiac enzymes (NT-proBNP, troponin), higher markers of inflammation (CRP, ferritin, D-dimer), and biochemical parameters such as high lactates, creatinine, low albumin, and sodium level, as well as lower concentration of PLT, leukocytes, and lymphocytes, are statistically significant factors for resistance to the administration of IVIG. Parameters that influenced the need to add systemic steroids to the treatment regimen were laboratory parameters: higher levels of NT-proBNP, troponin, creatinine, triglycerides, lactate, CRP, PCT, ferritin, and D-dimers; and low levels of lymphocytes, PLT, sodium, and albumin, as well as lower EF. In the performed statistical analysis, it was not found that none of the parameters significantly influenced the necessity to administer biological or immunosuppressive treatment.

In a large study conducted in the USA in 2020 by the Centers for Disease Control and Prevention, a database of 1080 patients meeting the PIMS-TS criteria was created. On the basis of the collected data, it was observed that ICU admission was more likely in patients aged 6–12 years, and more likely in non-Hispanic Black patients. ICU patients more often presented shortness of breath and abdominal pain, and the laboratory tests showed increased concentrations of CRP, troponin, ferritin, D-dimer, BNP, NT-proBNP, or interleukin-6, or reduced PLT or lymphocyte counts. Similar associations were stated for decreased cardiac function, shock, and myocarditis. Coronary artery abnormalities were more common in male patients and patients with mucocutaneous lesions or conjunctival injection. Obesity was linked to decreased cardiac function. The only laboratory markers associated with coronary artery abnormalities were NT-proBNP and interleukin-6 [8]. In our study group, decreased cardiac function was associated with increased concentrations of NT-proBNP, troponin, TG, ferritin, D-dimers, and creatinine and lower concentration of leukocytes, lymphocytes, PLT, albumin, and low-density lipoprotein. None of the tested laboratory parameters or demographic data influenced the risk of developing coronary aneurysms in the study group.

Another study that describes the risk factors for a more severe course PIMS-TS also found that the risk of admitting a child to ICU increases in children over 6 years of age. The study also showed that high levels of inflammatory markers, especially serum ferritin (> 500 mcg/ L), increase the risk of transfer to ICU by 18% [9]. In our study, group concentration of ferritin in a group of patients who required ICU admission was 1047 mcg/L vs 244 mcg/L in patients who did not require ICU admission. A more severe course of the disease was also found in children with changes in the skin and mucous membranes. The PIMS-TS cases identified later in the pandemic had a higher risk of admission to the ICU and of complications in the circulatory system. This proves that the mutating virus alters the body’s immune response to infection [9].

In a study conducted by Mól et al. efforts were made to isolate risk factors for significant changes in the circulatory system in children with PIMS-TS. The group with cardiac dysfunction or shock symptoms had significantly elevated markers of inflammation, granulocytosis, and significant lymphopenia, low levels of albumin and sodium, and significantly higher levels of NT-proBNP [10]. In our study group, children with hypotension and shock symptoms had significantly lower hematocrit, lymphocytes, and PLT and higher concentration of CRP, PCT, ferritin, D-dimers, TG, creatinine, NT-proBNP, and troponin I. In the study mentioned above, similar to ours, there were no differences in demographic parameters, fever duration, rash presence, or gastrointestinal symptoms in groups with and without cardiovascular involvement. However, a strong positive correlation was found between conjunctivitis and cardiac failure. It was also found that on the basis of laboratory tests alone, the severe course of the PIMS-TS syndrome can be excluded in only 20% of patients. In patients with elevated markers of inflammation and myocardial damage, such risk is difficult to estimate [10].

In the course of changes in the cardiovascular system and peripheral vasodilation, a significant proportion of patients develop hypotension and shock, which required hospitalization in ICU and the administration of catecholamines [1]. According to data from the UK, the number of admissions to pediatric ICU has increased 11 times since the onset of PIMS [11]. In our study group, 31% of children required a transfer to the ICU. Parameters correlating with the more severe course of the disease and a transfer of the child to the ICU in our study group are presented in Fig. 1.

None of the available studies presents any clear correlations between clinical features, laboratory tests, and treatment, with the risk of having coronary artery abnormalities or necessity of hospitalization in the ICU [1]. In the study describing risk factors of being admitted to the ICU for children with PIMS-TS, it was found that newborns, females, and children with comorbidities are more susceptible to a severe course of the disease. Decreased risk was stated in children aged 15–17 years [12].

The literature also raises the subject of obesity as a civilization disease of the current generation of children and adolescents, which also increases the risk of developing and severe course of both SARS-CoV-2 and PIMS-TS. It is recommended that obese patients should be closely monitored after contracting COVID-19 [13].

## Study limitations

The main limitation of the study is a relatively small group of patients, thus any outcomes of the study cannot be used as recommendations. Due to the novel nature of the disease, there are no large comparative studies. There is no long-term follow-up of the presented patients.

## Conclusions

In the course of our study, we showed that children with initially higher concentrations of NT-proBNP, troponin, creatinine, TG, CRP, PCT, ferritin, and D-dimers and lower hematocrit, PLT, lymphocytes, and EF should be strictly observed as they have a higher risk of severe course of the disease. Patients with high concentration of NT-proBNP, troponin, CRP, lactates, ferritin, D-dimers, and creatinine and a lower concentration of PLT, albumin, leukocytes, lymphopenia, and hyponatremia are at risk for immunoglobulin treatment resistance.

## Abbreviations

PIMS-TS: Pediatric multi-system inflammatory syndrome temporally associated with COVID-19
IVIG: Intravenous immunoglobulins
ICU: Intensive care unit
NT-proBNP: N-terminal pro-brain natriuretic peptide
TG: Triglycerides
PLT: Platelet count
CRP: C-reactive protein
PCT: Procalcitonin
EF: Ejection fraction
HDL: High-density lipoprotein
